# From Machine Learning to Ensemble Approaches: A Systematic Review of Mammogram Classification Methods

**DOI:** 10.3390/diagnostics15222829

**Published:** 2025-11-07

**Authors:** Hanifah Rahmi Fajrin, Se Dong Min

**Affiliations:** 1 Department of Software Convergence, Soon Chun Hyang University, Asan 31538, Republic of Korea; hanifah.fajrin@vokasi.umy.ac.id; 2 Department of Medical Electronics Technology, Universitas Muhammadiyah Yogyakarta, Yogyakarta 55183, Indonesia; 3 Department of Medical IT Engineering, Soon Chun Hyang University, Asan 31538, Republic of Korea

**Keywords:** breast cancer, mammogram classification, deep learning mammogram, machine learning mammogram, hybrid/ensemble mammogram

## Abstract

**Background/Objectives**: Breast cancer remains one of the leading causes of mortality among women, necessitating continued advancements in diagnostic methods to enhance early detection and treatment outcomes. This review explores the current landscape of breast cancer classification, focusing on machine learning (ML), deep learning (DL), and hybrid/ensemble models. **Methods**: A systematic search following PRISMA guidelines identified 50 eligible studies published between 2018 and 2025. Studies were included based on their use of mammogram datasets and implementation of computer-aided diagnosis methods for classification. Models were compared in terms of preprocessing, feature extraction, optimization strategies, and classification performance. **Results**: Representative high performing models illustrate the strengths and limitations of each approach. In ML, an optimized ELM achieved 100% accuracy on MIAS. DL methods such as Vision Transformers also reached 100% accuracy on DDSM, outperforming conventional CNNs. Hybrid models, particularly IEUNet++, achieved 99.87% accuracy, offering robust multi class classification. **Conclusions**: While ML and DL approaches can achieve near perfect accuracy, they typically focus on binary classification tasks and require extensive preprocessing, feature extraction, and optimization. In contrast, hybrid methods provide comparable or superior performance while simultaneously addressing multi-classification with fewer handcrafted steps, highlighting their robustness. These findings underscore the need for innovative solutions that balance model accuracy, interpretability, and resource efficiency. By addressing these challenges, future classification systems can better support early breast cancer detection and improve patient outcomes.

## 1. Introduction

Breast cancer continues to pose a major global health challenge, affecting millions of women each year and ranking among the leading causes of cancer-related deaths [1]. In 2020, the World Health Organization reported 2.3 million new cases of breast cancer and approximately 685,000 fatalities [2]. The critical role of early detection in improving patient outcomes cannot be overstated, as timely diagnosis can raise the survival rates to nearly 90% [3]. Considering this, the development of advanced diagnostic tools is essential to support clinicians in making accurate and timely decisions.

**Figure 1:**
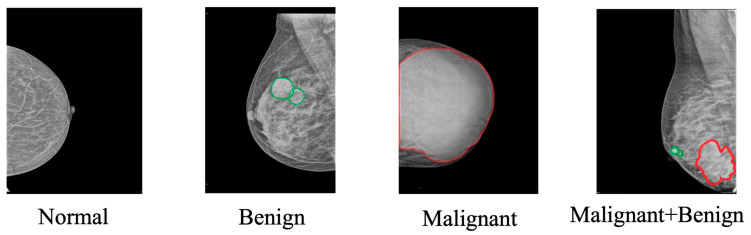
Classification of the breast. Green circles indicate benign regions of interest (RoI), and red circles delineate malignant lesions. The four panels illustrate the categories: Normal, Benign, Malignant, and Malignant + Benign.

Among the processes integral to breast cancer diagnostics is the classification of mammographic images, which involves distinguishing between normal, benign, malignant, and both benign and malignant tissues [4] (Figure 1). Effective classification aids in guiding clinical decisions for further examinations or treatments [5]. However, this task is not without its complexities. The diverse presentation of tumor characteristics, such as size, shape, and tissue density [6], poses significant challenges, particularly in cases involving dense breast tissue where visual differentiation is difficult [7].

To enhance the accuracy of classification and support radiologists, Computer-Aided Diagnosis (CAD) systems have become significant. These systems incorporate a range of classification methods that aim to improve diagnostic efficiency and reduce the likelihood of misinterpretation [8]. Traditional classification techniques, while foundational, often struggle with handling the heterogeneous nature of mammogram data. Machine learning (ML) approaches have addressed some of these limitations by enabling models to learn from data and adapt to complex patterns [9]. More recently, deep learning (DL) models, particularly convolutional neural networks (CNNs), have shown notable success in automating feature extraction and achieving high classification accuracy. Despite their promise, DL models can be limited by their need for large, well-annotated datasets and the significant computational power required for training [10]. In response to these challenges, hybrid and ensemble models have been explored as a means to optimize classification outcomes. By combining the strengths of traditional ML algorithms and DL architectures, hybrid/ensemble models aim to deliver enhanced accuracy and adaptability [11].

Previously, several review articles have addressed the application of machine learning and deep learning in breast cancer classification using mammograms [8,9,10,11,12,13]. For instance, refs. [9,10] provided overviews of ML/DL applications but did not explore ensemble learning in depth. Refs. [8,12] mainly focused on CAD systems, lacking comparative analysis across recent classification models. Other reviews such as refs. [11,13] offer broad discussions but do not highlight research gaps or trends in ensemble methods for mammogram classification. Unlike existing review papers that broadly cover breast cancer detection or combine various imaging modalities, this review focused exclusively on mammogram-based classification using machine learning, deep learning, and hybrid/ensemble methods. It proposes a structured taxonomy, highlights performance trade-offs, and critically compares the strengths and limitations of each approach. To the best of our knowledge, no prior review has offered such a focused and in-depth comparative analysis dedicated solely to classification techniques for mammogram images. The comparative analysis and clear taxonomy presented in this study can streamline model selection processes, support informed decision-making in computer-aided diagnosis systems, and foster further exploration in ensemble-based classification strategies. Accordingly, the primary contributions of this review can be summarized as follows:

1. An evaluation of a wide range of classification methods, from machine learning, deep learning to hybrid/ensemble models, applied specifically to breast cancer diagnosis using mammogram images, along with their integration into CAD systems.
2. Through a critical comparative analysis of recent works, the study highlights performance trends, trade-offs, and taxonomy that can assist researchers and practitioners in choosing appropriate models.
3. An exploration of limitations encountered in current research and practical implementation, followed by recommendations intended to guide future investigations and support the advancement of more effective detection tools.

The remainder of this review is structured as follows: Section 2 describes the methodology employed in this study, including the PRISMA framework and criteria for article selection. Section 3 presents a comprehensive overview of classification techniques used in mammogram analysis, including machine learning, deep learning, and hybrid or ensemble methods. Section 4 provides a critical discussion of the reviewed approaches, highlighting key insights, comparative observations, and gaps in the literature. Section 5 outlines the current challenges, potential opportunities, and future research directions in the field. Finally, Section 6 concludes the review by summarizing key findings and discussing their broader implications for research and clinical practice.

## 2. Materials and Methods

**Figure 2:**
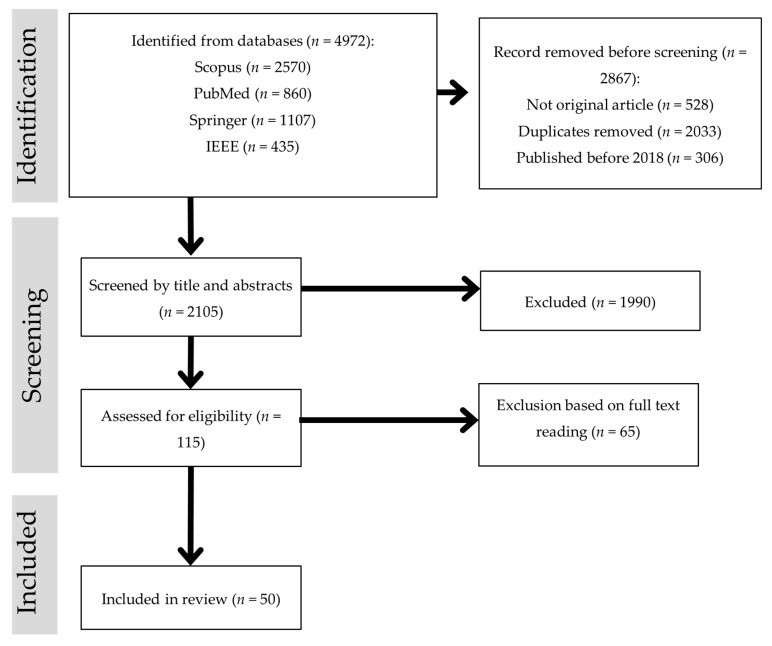
PRISMA flowchart of study selection. Gray bands indicate PRISMA stages; arrows show the flow of records; *n* = number of records.

This systematic review adhered to the PRISMA framework for a rigorous and transparent selection of relevant studies [14]. The PRISMA flow diagram (Figure 2) illustrates the three main stages: identification, screening, and inclusion.

1. Identification: A comprehensive search was conducted in four major databases—Scopus, PubMed, SpringerLink, and IEEE Xplore—to identify studies on mammogram-based computer-aided detection (CAD). The search covered publications from 2018 to 2025 using keywords such as “CAD for mammogram”, “mammogram classification”, “deep learning mammogram”, “machine learning mammogram”, and “hybrid/ensemble mammogram”. A total of 4972 records were retrieved (Scopus = 2570; PubMed = 860; SpringerLink = 1107; IEEE Xplore = 435). Before screening, 2867 records were removed, including 528 non-original articles (e.g., reviews, editorials), 2033 duplicates, and 306 studies published before 2018.
2. Screening and eligibility assessment: After initial removals, 2105 records underwent title and abstract screening to remove studies that were clearly irrelevant to mammogram-based segmentation or classification. This step excluded 1990 records. The remaining 115 studies were then subjected to a full-text eligibility assessment. Articles were excluded at this stage if they:
  - were non-journal publications (e.g., conference papers, book chapters),
  - lacked a primary focus on classification (e.g., preprocessing techniques, feature extraction/selection, segmentation, optimization algorithms),
  - used imaging modalities other than mammography,
  - were inaccessible due to paywalls, or
  - did not provide sufficient methodological or result details relevant to classification.

As a result, 65 full-text articles were excluded.

3. Inclusion: After applying these criteria, 50 studies were included in the review, offering insights into various classification techniques pertinent to mammogram-based breast cancer detection.

## 3. Results

**Figure 3:**
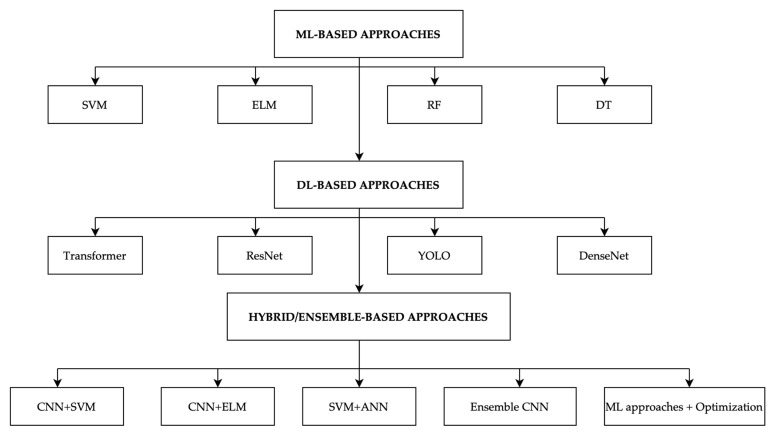
Taxonomy of classification methods in mammography.

In this section, we present the main findings of our systematic review on mammogram classification methods. The results are organized to highlight how different computational approaches, ranging from traditional machine learning (ML) to deep learning (DL) and hybrid/ensemble methods that have been applied in breast cancer detection. Each classification strategy has its own strengths in handling the unique challenges of breast cancer imaging, such as distinguishing subtle variations in tumor appearance and adapting to diverse imaging conditions [12]. To illustrate this categorization, Figure 3 presents a taxonomy of mammogram classification methods, where representative algorithms are shown for each group. For instance, ML-based approaches are represented by SVM, ELM, RF, and DT; DL-based approaches include CNN variants such as ResNet, DenseNet, YOLO, and Transformers; while hybrid/ensemble approaches combine models (e.g., CNN–SVM, CNN–ELM, or optimization-based ensembles). Following this taxonomy, the subsequent Section 3.1, Section 3.2 and Section 3.3 present a detailed discussion of ML, DL, and hybrid/ensemble-based classification approaches.

### 3.1. Machine Learning (ML)-Based Classification

Machine Learning (ML) has transformed breast cancer detection by allowing for the development of models that can learn patterns from mammogram data and classify images into some categories [15]. ML-based classifiers rely on advanced algorithms such as Support Vector Machines (SVM), Extreme Learning Machines (ELM), Random Forests (RF), and k-Nearest Neighbor (k-NN). A summary of machine learning-based classification methods, including their algorithms, datasets, and performance outcomes, is presented in Table 1.

Support Vector Machine (SVM) is one of the most widely used classifiers in breast cancer detection, and it has been employed by several researchers with varying results. Avcı & Karakaya [17] applied SVM to the MIAS dataset, using k-means clustering for segmentation and extracting texture features such as Gray-Level Co-occurrence Matrix (GLCM) and Gray-Level Run Length Matrix (GLRLM). Their model performed well in distinguishing benign from malignant tumors, although the small size of the MIAS dataset limited the generalizability of their results. Meanwhile, Ketabi et al. [18] working with the DDSM dataset, also employed SVM but used spectral clustering for segmentation and optimized their feature set using a Genetic Algorithm (GA), which reduced the feature set from 65 to 21. They achieved 90% accuracy but encountered difficulties with complex mass boundaries and overlapping tissues, limiting the model’s effectiveness in heterogeneous images. Sha et al. [19] adopted a different approach, using SVM classifier after feature extraction was conducted by Convolutional Neural Network (CNN) and optimizing the features with the Grasshopper Optimization Algorithm (GOA). Tested on the MIAS and DDSM datasets, this model achieved a high accuracy of 92%, with sensitivity and specificity both surpassing the results of [17]. However, the computational cost of the model in [19] was significantly higher due to the complexity of the optimization process.

**Figure 4:**
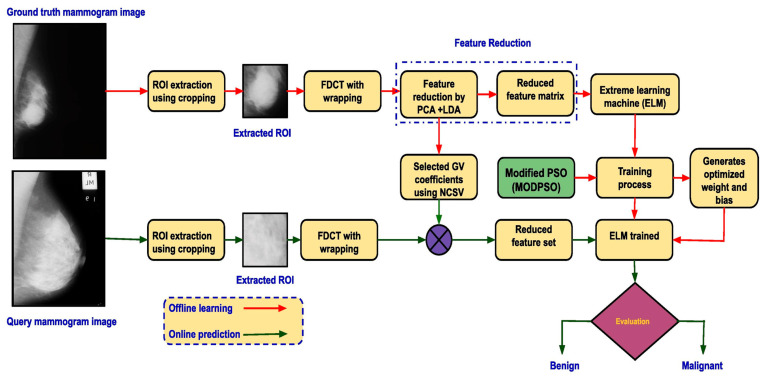
ELM-based classification [21]. Adapted from Muduli et al., “Fast discrete curvelet transform and modified PSO based improved evolutionary extreme learning machine for breast cancer detection”, Biomedical Signal Processing and Control, Volume 70, September 2021, 102919, with the permission from Elsevier. Copyright © 2021 Elsevier.

Another popular ML classifier is the Extreme Learning Machine (ELM), known for its efficiency in training large datasets. Both Wang et al. (2019) [16] and Muduli et al. (2021) [21] used ELM for classifying breast masses, but they approached feature extraction and model design differently. Wang et al. [16] used ELM on a private dataset of 400 mammograms, extracting features using a CNN, which focused on the morphology, texture, and density of breast masses. ELM achieved a classification accuracy of 96.2%, with high sensitivity and specificity, though the study noted that manual feature extraction introduced variability depending on expert input [16]. Muduli et al. [21] adopted a more complex framework, integrating Particle Swarm Optimization (PSO) with ELM, which was tested on the MIAS, DDSM, and INbreast datasets. Their model achieved even higher accuracies of 98.94% on MIAS and 98.76% on DDSM and INbreast due to the combination of Fast Discrete Curvelet Transform (FDCT) for feature extraction and PCA for dimensionality reduction; this research’s scheme can be seen in Figure 4. However, the complexity of this model and its high computational cost were noted as significant drawbacks, particularly for large-scale clinical implementation [21].

Random Forest (RF) is another widely used ML classifier in mammogram classification due to its robustness and ability to handle large datasets. Avcı and Karakaya [17] tested RF on the MIAS dataset, alongside other classifiers like SVM and k-Nearest Neighbor (k-NN), after segmenting the images using k-means clustering and extracting texture-based features such as GLCM and GLRLM. RF showed competitive performance compared to SVM, and while the MIAS dataset’s limited size impacted the model’s ability to generalize, the RF approach proved useful in managing feature variability. Thawkar & Ingolikar [22] applied RF to the DDSM dataset, using morphological and texture-based features for classification, and achieved an accuracy of 94.6%. RF’s ability to handle complex datasets without overfitting made it a valuable tool in this context, though the need for large, diverse datasets remains a limitation in ensuring robust generalization.

k-Nearest Neighbor (k-NN), though simpler than SVM or RF, has also been explored as an effective ML classifier. Sannasi et al. [20] applied Weighted k-NN (wKNN) to the MIAS and INbreast datasets, achieving an accuracy of 84.35% on MIAS and 83.19% on INbreast. To optimize performance, they used metaheuristic algorithms such as Particle Swarm Optimization (PSO), Dragonfly Optimization Algorithm (DFOA), and Crow-Search Optimization Algorithm (CSOA). Although k-NN’s simplicity is an advantage, the model’s performance was heavily influenced by the choice of optimization algorithm. DFOA required significant parameter tuning and showed slower convergence compared to PSO, increasing the model’s computational cost in classification. Finally, Decision Trees (DT) have been explored as a simpler, interpretable classification method. Thawkar and Ingolikar (2020) employed a decision tree model on the DDSM dataset, achieving an accuracy of 92.7%. Although decision trees provide transparency and are easy to interpret, they are prone to overfitting, particularly on small datasets [25].

### 3.2. Deep Learning (DL)-Based Classification

Deep learning (DL) methods have revolutionized mammogram classification, leveraging neural networks to automatically learn hierarchical features from images. An overview of deep learning models used in breast cancer classification can be found in Table 2.

Convolutional Neural Networks (CNNs) have been widely adopted for mammogram classification, with several studies utilizing different architectures. Han et al. (2024) [28], Liu et al. (2022) [30], and Shu et al. (2020) [31] all employed CNN-based models with DenseNet architectures for feature extraction. Han et al. [28] proposed a Deep Location Soft-Embedding-Based Network (DLSEN-RS), applying it to the CBIS-DDSM and INbreast datasets. The model achieved high accuracy, with an AUC of 0.962 and an accuracy of 91.5% for INbreast, and an AUC of 0.948 and accuracy of 89.4% for CBIS-DDSM. However, one limitation noted was the difficulty in determining the optimal number of features (k), which could impact model performance if not selected properly. Liu et al. [30] introduced a Deep Multiscale Multi-Instance Network for classification, also utilizing DenseNet for feature extraction. On the INbreast dataset, the model achieved an AUC of 0.975 and accuracy of 93.2%, outperforming Han’s model slightly, though the challenge of selecting optimal k values also persisted here. Shu et al. developed a Deep Neural Network with Region-Based Pooling and applied it to both the INbreast and CBIS-DDSM datasets, achieving an AUC of 0.982 and accuracy of 91.6% on INbreast, and an AUC of 0.882 with an accuracy of 83.9% on CBIS-DDSM. Shu’s model focused on region-based pooling, but this technique significantly increased the processing time and computational resource requirements, which may hinder real-time applications [31].

Another significant contribution came from Nasir Khan et al. (2019) [33], who proposed a Multi-View Feature Fusion Model using various CNN architectures, including VGGNet, ResNet, and GoogLeNet, to classify mammogram images from the MIAS and CBIS-DDSM datasets. This multi-view approach incorporated images from different angles of the breast, achieving an AUC of 0.932 for detecting masses and calcifications, and an AUC of 0.84 for distinguishing between malignant and benign cases [33]. However, transfer learning is another approach that has gained popularity in mammogram classification. Le et al. (2024) applied ResNet-34, pre-trained on ImageNet, to classify images from the DDSM and Hanoi Medical University (HMU) datasets. With transfer learning, they achieved a macro-AUC of 0.766 on the HMU dataset. Although transfer learning allowed them to leverage pre-trained networks for faster convergence and higher performance, the availability of annotated mammogram datasets remained a limitation, affecting the fine-tuning process and overall generalizability of the model [34].

**Figure 5:**
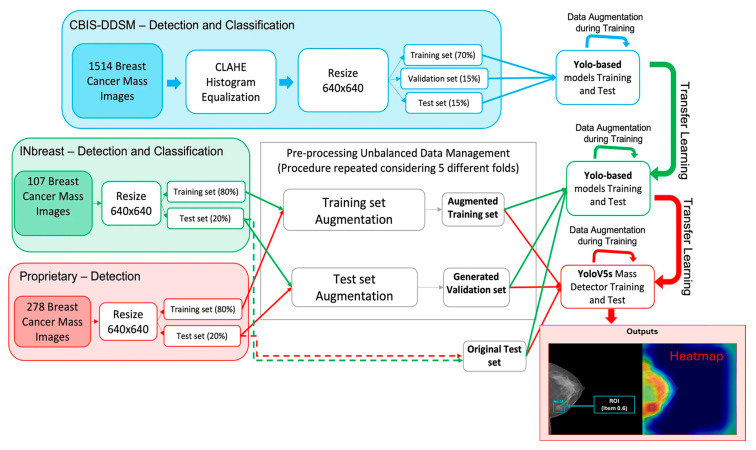
A YOLO-BasedModel for Breast cancer detection [50]. Legend: blue arrows = data split to YOLObased models (train/val/test); red arrows = training/augmentation flow; green arrows = test/inference flow; dashed red/green = cross-set links; dashed blue box = feature-reduction block; cyan call-out = detected ROI. Adapted from Prinzi et al., “A YOLO-BasedModel for Breast Cancer Detection inMammograms”, Cognitive Computation, Volume 16, pages 107–120, 2024. Copyright © 2023 by the authors. Published by SpringerNature under a Creative Commons Attribution License, with the output example image (bottom right) modified for clarity.

YOLO models have also been adapted for mammogram classification. Anas et al. (2024) [29] applied an enhanced YOLOv5 network combined with Mask R-CNN for classification on the INbreast, CBIS-DDSM, and BNS datasets, reporting a false positive rate (FPR) of 0.049% and a false negative rate (FNR) of 0.029%. The model also achieved an impressive Matthews Correlation Coefficient (MCC) of 92.02%, although the computational complexity of training two networks (YOLOv5 and Mask R-CNN) simultaneously was highlighted as a major limitation. Meanwhile, ref. [50] utilized YOLOv5 for lesion detection and classification on the CBIS-DDSM and INbreast datasets, achieving a mean Average Precision (mAP) of 0.835 on INbreast and 0.498 on CBIS-DDSM (the flowchart can be seen in Figure 5). While the results were promising, the YOLO model tended to be biased toward smaller lesions, potentially missing larger, more complex tumors.

### 3.3. Hybrid/Ensemble Classification Methods

Ensemble/hybrid techniques combine multiple classifiers or integrate different machine learning models to leverage their strengths, thereby enhancing classification performance [51]. Table 3 provides an overview of method used in hybrid/ensemble approaches.

SVM and CNN combinations are a common hybrid approach employed to enhance classification performance. Ahmad et al. [52] developed a hybrid model called BreastNet-SVM, which combines a modified AlexNet CNN and an SVM classifier for final classification. Applied to the DDSM dataset, this model achieved an impressive accuracy of 99.16%, with a sensitivity of 97.13% and a specificity of 99.30%. Despite these high results, the performance of the model was sensitive to the choice of optimizers and hyperparameter tuning, which could affect the generalizability of the model. Similarly, ref. [55] combined a CNN with ELM classifier for breast cancer detection on the MIAS dataset, achieving an accuracy of 86%. However, the study emphasized the need for validation on larger and more diverse datasets. Furthermore, in the study conducted by [61], the authors explored a comparison between standalone SVM and ANN methods versus their hybrid model, SVM-ANN, for classifying mammogram images. Using the Mini-MIAS dataset, which consisted of 80 normal, 40 benign, and 40 malignant mammograms, the researchers found that standalone SVM achieved a classification accuracy of 78.8% for distinguishing normal from abnormal cases and 71.3% for benign versus malignant. The ANN, on the other hand, performed slightly better with 83.1% accuracy for normal/abnormal classification and 78.8% for benign/malignant. Notably, the hybrid SVM-ANN model significantly outperformed both standalone methods, achieving an impressive 99.4% accuracy for normal versus abnormal.

Another approach integrating ensemble learning with feature weighting algorithms was proposed by [54]. They applied an ensemble model consisting of k-Nearest Neighbor (k-NN), bagging, and EigenClass algorithms, using a majority voting rule for classification. Their model was applied to both the MIAS and DDSM datasets, achieving an accuracy of 93.26% on DDSM and 91% on MIAS. This ensemble model benefited from the diversity of classifiers, but the computational complexity introduced by both the ensemble framework and feature weighting algorithms posed challenges, particularly in terms of processing time. Several studies also explored hybrid models combining optimization algorithms with classifiers. Muduli et al. (2020) [67] proposed a hybrid Moth Flame Optimization (MFO)-ELM model that combined the ELM classifier with the MFO algorithm to optimize the hidden layer weights and biases. Applied to both the MIAS and DDSM datasets, the model achieved excellent performance, with an accuracy of 99.76% for normal vs. abnormal classification and 98.80% for benign vs. malignant classification on the MIAS dataset. Despite these impressive results, the random initialization of ELM parameters occasionally introduced instability, which could affect the model’s reliability, though this was mitigated by the optimization algorithm.

**Figure 6:**
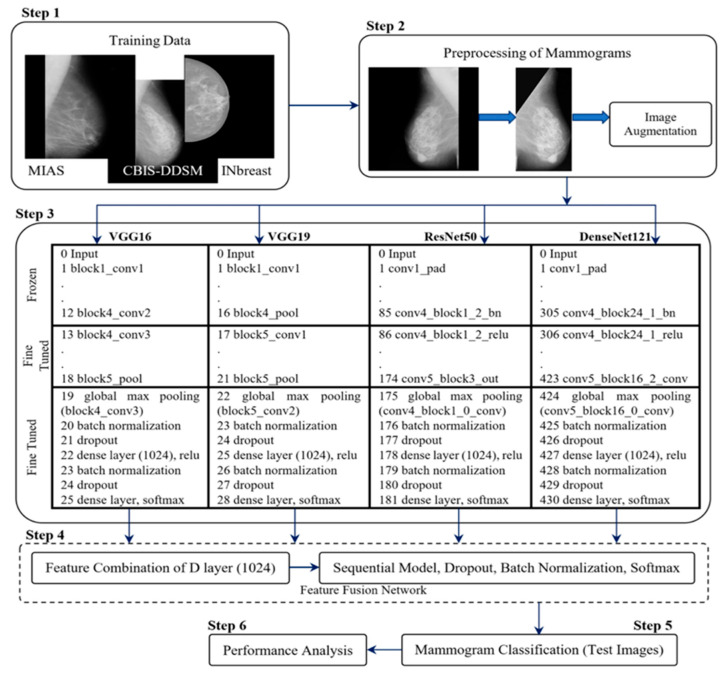
The model of CNN combination [57]. Reprinted from Chakravarthy et al., “Multi-class Breast Cancer Classification Using CNN Features Hybridization”, International Journal of Computational Intelligence Systems, 17, 191 (2024). Copyright © 2024 by the authors. Published by Springer Nature under a Creative Commons Attribution 4.0 License.

Kalpana and Selvy (2024) [56] also utilized hybrid/ensemble techniques, proposing an ensemble model combining Naïve Bayes, Firefly Binary Grey Optimization (FBGO), and a Transfer-CNN (TCNN) coupled with Moth Flame Lion Optimization (MMFLO). Applied to the MIAS, INbreast, and BCDR datasets, the model achieved an accuracy of 96.3% with Naïve Bayes and 98% with TCNN. The study highlighted the complexity of combining multiple classifiers, with the computational load increasing significantly when blending Naïve Bayes with TCNN. Nevertheless, the ensemble model’s ability to perform well across multiple datasets demonstrated its versatility. In another study, Chakravarthy et al. (2024) [57] applied a hybrid approach by combining features extracted from four different CNN architectures (VGG16, VGG19, ResNet50, and DenseNet121) and merging them for final classification (the ensemble flowchart can be found in Figure 6). Tested on the MIAS, CBIS-DDSM, and INbreast datasets, their model achieved accuracy rates of 98.70% on MIAS, 97.73% on CBIS-DDSM, and 98.83% on INbreast. However, this approach introduced computational complexity due to the combination of multiple CNN models. The study also noted slight difficulties in discriminating between malignant and benign cases compared to normal cases, indicating that further refinement of the hybrid approach may be necessary for improving the classification of malignant cases. Lastly, ref. [60] investigated quantum transfer learning for breast cancer detection, applying a hybrid classical-quantum model that combined traditional neural networks with quantum enhancements. The study utilized the BCDR dataset, which consists of mammogram images categorized as benign or malignant. The proposed approach incorporated a quantum circuit attached to a pre-trained ResNet18 model, acting as a feature extractor while the quantum circuit performed the classification task. The results showed an accuracy of 84%, outperforming the classical standalone approach, which achieved 67% accuracy. The comparison highlighted that the hybrid classical-quantum model demonstrated improved generalization and faster convergence.

## 4. Discussion

**Figure 7:**
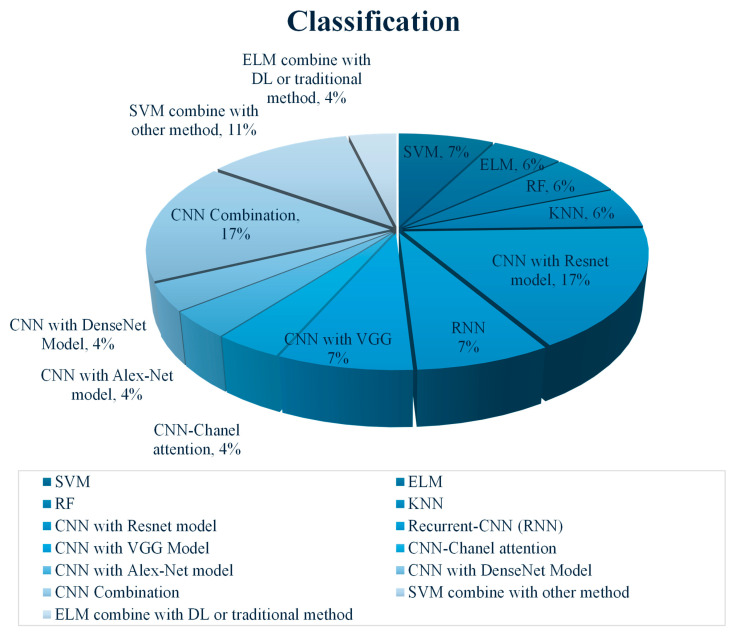
Distribution of classifier.

Figure 7 illustrates the distribution of classification methods based on references in this article. In breast cancer classification, ResNet and Recurrent Neural Network (RNN), as deep learning models, represented a significant portion of studies. ResNet’s deep architecture, especially ResNet152V2, excels in feature extraction and classification due to its residual connections, which capture complex patterns in mammograms. For example, ref. [43] utilized a ResNet152V2-based approach within a three-step framework, achieving perfect accuracy for breast density and tumor malignancy classification, while RNNs enhanced temporal data handling, making them particularly useful for tracking changes across mammogram slices, with an accuracy of 98% for tumor classification (benign and malignant).

Other deep learning CNN architectures, including VGG, Channel Attention, AlexNet, and DenseNet, remain widely used in breast cancer classification due to their unique strengths in feature extraction and image analysis. VGG, appearing in 8% of studies, is favored for its straightforward yet deep structure, enabling detailed feature extraction with manageable computational demands, ideal for varied research settings. Channel Attention, used in 4% of studies, enhances classification accuracy by focusing on critical regions in mammograms, such as calcifications or tumor borders, which are diagnostically significant. AlexNet and DenseNet, each also employed in 4% of studies, contribute hierarchical and densely connected layers, respectively, to improve feature propagation in complex analyses. AlexNet’s multi-layered feature extraction is effective for capturing various levels of detail, while DenseNet’s connectivity facilitates the learning of intricate patterns, especially in mammograms with subtle or complex textures.

Meanwhile, hybrid/ensemble approaches prove effective by combining CNNs and SVMs. In one example, ref. [59] integrated EfficientNet-B7 and ConvNeXt-101, achieving high AUC scores (up to 0.98) across multiple datasets. This method allows for adaptable feature representation across diverse textures in mammograms, reducing false positives and ensuring reliable diagnostic results. SVM Combination methods, representing 11% of studies, are used to leverage SVM’s effectiveness in binary classification along with deep learning models for feature extraction. Ahmad et al. (2023) combined SVM with AlexNet model, achieving an accuracy of 99.16% on the DDSM dataset for benign versus malignant classification [52]. This hybrid approach combines the precision of SVM with the comprehensive feature extraction of CNNs, improving overall diagnostic accuracy.

The standalone SVM machine learning approach, widely used by researchers in around 8% of studies, remains a reliable option for straightforward classification tasks. Sha et al. (2020) utilized SVM on the MIAS and DDSM datasets, achieving 92% accuracy. SVM’s simplicity makes it a suitable choice for smaller datasets where classes are well-separated [19]. Other Machine learning-based methods, ELM, RF, and KNN, each represented 6% of studies. Mohanty et al. (2020) [24] implemented ELM, achieving over 99% accuracy across the MIAS, DDSM, and BCDR datasets. ELM’s fast training makes it suitable for scenarios requiring rapid classification. Random Forest, which builds multiple decision trees to reduce overfitting, is effective for complex datasets, while KNN provides a simple, yet robust classification based on nearest-neighbor analysis, ideal for binary tasks.

All in all, these methods reflect the diversity of classification techniques in breast cancer detection. ML models like SVM remain practical for well-defined classification tasks [68], while deep learning architectures such as ResNet and VGG excel in recognizing intricate textures [69]. Hybrid approaches, combining strengths from different models, provide adaptable solutions across varied mammographic imaging challenges, ensuring an optimal balance between accuracy and efficiency. The inclusion of feature extraction and optimization techniques further boosts classification accuracy, as seen in the studies by [56,67]. For example, Naïve Bayes combined with Firefly Binary Grey Optimization (FBGO) achieved a 96.3% accuracy on the MIAS dataset, and TCNN combined with Moth Flame Lion Optimization (MMFLO) achieved 98%, underscoring the impact of optimizing classifier parameters for improved sensitivity and specificity [56]. Similarly, MFO-ELM, which integrates Lifting Wavelet Transform for feature extraction and optimizes ELM parameters, achieved near-perfect classification on the MIAS dataset with 99.76% accuracy for normal vs. abnormal cases, illustrating how optimized models refine feature capture and classification accuracy across diverse mammographic features [67]. Although computationally intensive, these optimizations are valuable for tasks requiring fine-tuned parameter adjustments in complex images (Table 4).

An in-depth comparative analysis of classification performance in breast cancer detection highlighted significant variations across machine learning, deep learning, and hybrid/ensemble approaches. As summarized in Table 4, machine learning (ML) models demonstrated accuracy levels ranging from 82.42% to 100%, with sensitivity reaching up to 99.1% and specificity extending to 98.72%. These results confirm ML’s reliability, particularly in binary classification tasks (e.g., normal vs. abnormal), when paired with well-extracted statistical features and classifiers such as Support Vector Machine or Random Forest. However, while the precision ranges between 82.42% and 83.87%, ML-based methods may underperform when dealing with class imbalance or subtle radiological variations in dense tissue regions.

Deep learning (DL) architectures exhibited an even broader accuracy range, between 70% and 100%, but often surpassed ML in terms of precision (up to 99.16%). Their strength lies in automated hierarchical feature extraction, allowing them to detect abstract imaging patterns that traditional models may overlook. Nonetheless, dependency on large, annotated datasets and high computational demands can hinder their adaptability in clinical settings with limited resources.

Interestingly, hybrid models that integrated the strengths of both ML and DL techniques achieved balanced and consistently high performance across the evaluation metrics. With the accuracy ranging from 74.96% to 99.87%, sensitivity from 96.2% to 97.77%, and specificity up to 99.8%, these models offer improved robustness, especially in multi-class classification scenarios (normal, benign, and malignant). Their ability to combine precise feature extraction with optimized decision-making layers makes them particularly suitable for complex mammographic analysis.

Table 5 presents a representative comparison of the top-performing methods across three main classification approaches: Machine Learning (ML), Deep Learning (DL), and Hybrid models. This table highlights a single, best-performing method within each category based on classification accuracy and reported evaluation metrics. This approach allows for a focused comparison of preprocessing steps, feature extraction/selection, optimization, and classification strategies employed by each method. In the ML-based approach proposed by [21], classification begins with extracting features from mammogram images using the Fast Discrete Curvelet Transform (FDCT), which is well-suited for capturing edge and texture details. To reduce redundancy and retain only the most discriminative information, dimensionality reduction is applied through Principal Component Analysis (PCA) and Linear Discriminant Analysis (LDA). These processed features are then classified using an optimized version of Extreme Learning Machine (ELM), enhanced by a Modified Particle Swarm Optimization (MODPSO) algorithm. MODPSO dynamically adjusts the input weights and biases of ELM, addressing common limitations such as instability and overfitting. This optimization process significantly improves the classifier’s ability to converge quickly and generalize well across datasets. The method achieved exceptional accuracy, notably 100% on the MIAS dataset, and above 98% on the DDSM and INbreast datasets. However, this pipeline involves several complex stages—including handcrafted feature extraction, selection, and optimization—making it computationally demanding. Furthermore, it only addresses binary classification (benign vs. malignant), limiting its utility in multi-class diagnostic scenarios.

Meanwhile, for deep learning (DL) approaches, a perfect classification performance (100% accuracy, AUC, and F1-score) was achieved in distinguishing between benign and malignant cases. This result was obtained through the implementation of Vision Transformer (ViT) architectures, including Swin Transformer and Pyramid Vision Transformer (PVT). These models were trained using a transfer learning strategy, where pre-trained weights from ImageNet were fine-tuned on the DDSM mammography dataset. Unlike CNNs that rely on sequential, localized feature extraction, ViT treats the image as a sequence of patches, allowing for global contextual understanding from early layers, enhanced by self-attention mechanisms and positional embeddings. However, this approach also depends heavily on preprocessing techniques and data augmentation strategies to mitigate class imbalance. In comparison, traditional CNN-based models such as ResNet18 and EfficientNetB0 achieved lower performance, with AUC values ranging between 0.80 to 0.85 and accuracy scores between 90% to 95%, indicating the superior generalization and precision of ViT-based models for binary classification [42].

In the hybrid approach by [66], the authors proposed IEUNet++, a novel deep hybrid model that integrates InceptionResNet, EfficientNetB7, and a U-Net-based segmentation backbone. Unlike traditional ensemble strategies that combine independent CNN models through fuzzy or voting mechanisms, IEUNet++ leverages a unified encoder–decoder architecture with multi-scale feature fusion. This design allows for the simultaneous segmentation and classification of mammogram images, capturing both local lesion details and global contextual features. The model was evaluated on the MIAS, CBIS-DDSM, and INbreast datasets, achieving exceptionally high performance with an accuracy of 99.87% across normal, benign, and malignant categories. Compared to conventional CNN classifiers, IEUNet++ demonstrated superior robustness by reducing feature redundancy and enhancing the discriminative capacity for subtle lesion patterns.

These findings collectively show that while ML and DL approaches can yield excellent results, they often require extensive preprocessing, feature engineering, or optimization to reach their full potential—and are typically limited to binary classification tasks. In contrast, the hybrid approach demonstrated not only superior performance without additional optimization techniques, but also the capacity to effectively handle multi-class classification scenarios, making it a compelling candidate for broader clinical deployment.

Despite these advances in model performance, the reliability and generalizability of classification systems also depend on the datasets used for training and validation. Public mammogram datasets have therefore played a central role in enabling benchmarking, comparison across methods, and the reproducibility of research findings.

Several public datasets have been extensively employed for mammogram classification tasks, as summarized in Table 6. Among them, the MIAS dataset (https://www.repository.cam.ac.uk/items/b6a97f0c-3b9b-40ad-8f18-3d121eef1459) (accessed on 12 February 2025) remains the most frequently used benchmark, despite its relatively small size of 322 images, which limits generalization. The DDSM dataset provides over 10,000 mammograms, making it one of the largest available, although its older image quality poses challenges for modern algorithms. To address these issues, CBIS-DDSM (https://www.cancerimagingarchive.net/collection/cbis-ddsm/) (accessed on 12 February 2025), a curated subset of DDSM with improved annotations, has become popular in recent studies. The INbreast dataset (https://www.kaggle.com/datasets/ramanathansp20/inbreast-dataset) (accessed on 12 February 2025), while small (410 images), is highly valued for its high-quality, pixel-level annotations. Lastly, the BCDR dataset (https://service.tib.eu/ldmservice/dataset/bcdr) (accessed on 12 February 2025) offers region-of-interest annotations and a moderate image size, though it is less frequently adopted compared to MIAS and DDSM. Overall, these datasets form the foundation of most mammogram classification research, with MIAS and DDSM dominating usage, while INbreast and CBIS-DDSM provide higher-quality but smaller-scale alternatives. In the table, “All kind” refers to datasets that include a variety of lesion types commonly found in mammography, such as masses, microcalcifications, architectural distortions, and asymmetries. This diversity makes them particularly useful for developing models that can generalize across different manifestations of breast cancer, rather than being restricted to only one lesion type.

However, many reviewed studies achieved high accuracy while still relying heavily on small or older datasets such as MIAS and INbreast. These datasets, although valuable for benchmarking, present several technical limitations. Their limited sample sizes, imbalanced datasets [37,45], and homogeneous image characteristics increase the risk of overfitting [61,62], making the models less reliable when tested on new or heterogeneous populations [17,27]. Moreover, the narrow diversity in breast density, lesion appearance, and imaging conditions reduces the model’s capacity to learn generalized features, while the outdated quality of older datasets like DDSM introduces additional domain gaps compared to modern clinical images [18,47]. As several studies in this review acknowledged, these factors can lead to an inflated or dataset-specific performance that does not translate effectively to clinical settings. Therefore, future research should emphasize validation using larger, multi-institutional, and demographically diverse datasets, as well as cross-dataset and domain-shift evaluations to ensure robust and clinically meaningful performance, for instance, by utilizing newer datasets such as VinDr-Mammo (20,000 images) [70] and RSNA (over 50,000 images) [71], which provide more recent, high-resolution mammograms with standardized DICOM formats and comprehensive annotations.

## 5. Challenges, Opportunities, and Future Directions in Breast Cancer Detection

Breast cancer detection using mammograms presents numerous challenges across classification stages. As methods evolve from machine learning (ML)-based, deep learning (DL)-based, and hybrid/ensemble approaches, each step brings its own set of difficulties that can limit the effectiveness, scalability, and clinical adoption of these techniques. Based on the methods discussed in Section 3 and Section 4, this Section 5 explores the primary challenges faced in classification, and general aspects of breast cancer detection.

### 5.1. Classification Challenges

The challenges faced in classification, whether using ML-based, DL-based, or hybrid/ensemble models, are significant and closely tied to the success of segmentation. Key issues persist despite advancements in model development:

1. Feature Extraction: Classification models heavily depend on the quality of feature extraction [72,73]. Classifiers such as SVM and Decision Trees (DTs) rely on manual feature extraction techniques like GLCM or HoG, which may not capture the full complexity of tumor characteristics [74,75]. Even in DL models, where features are automatically learned, extracting meaningful features from small or low-contrast tumors remains a challenge [19,31].
2. Overfitting: Overfitting is a common issue in ML and DL classifiers, particularly when models are trained on small or imbalanced datasets like MIAS or INbreast [21,28]. Models such as SVM, ELM, and even advanced CNN-based classifiers tend to perform well on training data but often fail to generalize to new, unseen data [16,17]. Hybrid models that combine multiple classifiers also risk overfitting when trained on small datasets [57].
3. Computational resources and time: DL models and hybrid approaches often require significant computational resources for training and inference. Models such as YOLO combined with Mask R-CNN or DenseNet architectures are computationally expensive and may not be feasible for real-time clinical applications [29,30]. Moreover, hybrid approaches that combine optimization algorithms with classification models, such as MODPSO-ELM, can further increase training times, limiting clinical implementation [21].

### 5.2. Opportunities and Future Direction of Breast Cancer Detection

This following subsection outlines key strategies that hold potential for enhancing breast cancer detection systems, emphasizing improvements in feature extraction, optimization, and model adaptability. By exploring these strategies, researchers can refine existing methods, adapt to varying imaging conditions, and facilitate real-time clinical applications.

1. Combining Feature Extraction with Classification: Hybrid models that integrate DL-based feature extraction (e.g., using CNNs) with ML classifiers, such as SVM and Random Forest (RF), have shown notable improvements in classification performance [52,55]. This fusion allows for better utilization of the learned hierarchical features from DL models, while ML classifiers can handle the final decision-making step. In studies like those by [21,56], hybrid models significantly boosted the accuracy in both segmentation and classification. Future research should explore more efficient combinations of these models and identify which pairing yields the best results under varying conditions.
2. Optimization Algorithms and Metaheuristics: Many hybrid methods include the use of metaheuristic algorithms [76], such as Particle Swarm Optimization (PSO), Genetic Algorithms (GA), or Moth Flame Optimization (MFO), to optimize model parameters and enhance performance [67]. These algorithms have proven effective in tuning model weights and improving the learning process [77], particularly when dealing with complex datasets like MIAS, DDSM, and INbreast. Future work should focus on integrating more sophisticated optimization techniques, such as reinforcement learning [78] or evolutionary algorithms, to further refine breast cancer detection models.
3. Transfer Learning: Transfer learning has emerged as a key opportunity for leveraging pre-trained DL models, such as ResNet, DenseNet, and EfficientNet, to reduce the computational burden associated with training deep models from scratch [34]. These models, trained on large datasets like ImageNet, can be fine-tuned [69] for specific breast cancer detection tasks [79], which allows researchers to overcome the challenges of limited mammogram datasets. Transfer learning has shown promise in improving classification accuracy while reducing the training time. Beyond efficiency, transfer learning also improves representation quality. For example, ResNet/DenseNet/EfficientNet backbones preserve fine-grained image details through skip connections and multi-scale feature extraction layers [80], which is particularly beneficial for subtle or low-contrast mammographic lesions. By reusing pretrained convolutional filters that already capture edge, gradient, and texture patterns, the model can enhance lesion visibility even when intensity differences are minimal. Fine-tuning only the higher-level layers allows for adaptation to mammography’s domain characteristics, such as glandular tissue density and microcalcification patterns, while maintaining robust low-level representations learned from large-scale datasets. Accordingly, future directions should involve exploring more domain-specific pre-training techniques that are tailored to medical images [81], ensuring that models are better suited to the nuances of mammogram data.
4. Integration with Other Imaging Modalities: Another promising direction for future research is the integration of mammogram analysis with other imaging modalities [82], such as ultrasound and MRI. Combining information from multiple imaging [83] techniques could improve the accuracy and robustness of breast cancer detection models by providing complementary views of the same region, reducing the likelihood of false negatives [84]. Such multimodal fusion is especially valuable for subtle or low-contrast lesions that are difficult to identify on mammograms alone, as ultrasound and MRI provide richer tissue contrast, margin definition feature, and contextual cues that help delineate ambiguous structures [84,85,86]. Beyond imaging, multimodal learning can also combine mammograms with clinical records, pathology reports, or other tabular data, enabling richer feature representation and potentially improving diagnostic performance. Recent works [87] have shown that integrating imaging with structured clinical data enhances model generalization and supports more clinically relevant decision-making.
5. Real-time Application and Model Efficiency: A major future goal is to develop models that can be deployed in real-time clinical environments. Techniques such as model pruning [88], quantization, and knowledge distillation can be explored to reduce the size and computational requirements of deep learning models without sacrificing accuracy. These methods will be essential for integrating AI-driven breast cancer detection systems into everyday clinical workflows, especially in under-resourced healthcare settings. Recent studies have shown that pruning removes redundant connections, and quantization reduces precision from 32-bit to lower bit widths (e.g., INT8), significantly decreasing the inference latency and energy consumption without major accuracy loss [89,90,91].
6. Clinical Perspective and Translation: Beyond technical performance, clinical adoption is essential for breast cancer classification models. For real-world use, models must provide interpretability, reliability, and validation across diverse patient populations and imaging protocols. Interpretability can be supported through visualization tools such as Grad-CAM [36] or attention heatmaps [53], which help radiologists understand the model’s decision basis. Meanwhile, clinical reliability requires rigorous external validation using independent and multi-institutional datasets to verify robustness beyond training conditions [92]. In practice, this involves testing models on data from different hospitals or imaging devices, reporting results at clinically relevant operating points (e.g., maintaining high sensitivity with corresponding specificity), and evaluating whether AI assistance improves radiologist performance or reading efficiency. Difficult or uncertain cases should be referred to for manual review rather than automated decision-making. Finally, practical deployment also depends on the clear reporting of inference time, hardware needs, and integration into daily clinical workflow [93]. Integration into radiology practice further requires efficiency, regulatory approval, and minimization of false positives to ensure radiologist trust. While current systems show promising accuracy, most remain at the proof-of-concept stage, emphasizing the need for large-scale validation and collaboration between engineers and healthcare professionals [94].
7. Foundation and Large Vision Models: Although transfer learning models such as ResNet, DenseNet, and EfficientNet have demonstrated strong performance by reusing pretrained representations from large-scale datasets, recent advancements have shifted toward foundation models and large vision models (LVMs) that offer broader generalization and adaptability. Foundation models are large-scale deep architectures trained on massive and diverse images or multimodal datasets, enabling them to serve as general-purpose backbones that can be adapted to various medical imaging tasks with minimal fine-tuning. In medical imaging, LVMs such as Vision Transformers (ViT) [42], Segment Anything Model (SAM) [95], MedCLIP [96], and BioViL have shown strong potential in capturing fine-grained anatomical patterns, handling cross-domain variations, and linking visual features with textual clinical information. These models not only provide richer visual-semantic representations but also allow zero-shot or few-shot adaptation, which is particularly beneficial when labeled medical data are limited. Applying such models to mammography could improve lesion localization and classification performance by leveraging their multi-scale and multimodal understanding. Future research should explore how foundation and large vision models can be effectively fine-tuned, compressed, or adapted for mammographic imaging, balancing their computational cost with clinical feasibility while maintaining interpretability and reliability for real-world deployment.

## 6. Conclusions

The advancement of breast cancer classification has evolved significantly through machine learning (ML), deep learning (DL), and hybrid/ensemble approaches, each offering distinct strengths and facing unique challenges. ML models, including popular classifiers like SVM, RF, and ELM, remain effective, particularly when combined with feature extraction and optimization techniques, but they rely heavily on careful feature selection to avoid performance degradation. Deep learning models, notably CNN architectures and vision transformers, excel through automatic feature extraction and robust performance, but they are typically resource-intensive and sensitive to data quality and imbalance. Hybrid and ensemble methods integrate multiple classifiers and diverse learning strategies, achieving improved accuracy and robustness, even though increased complexity and computational requirements could hinder clinical applicability. This review highlighted that each category of classification methods presents specific advantages and limitations. For instance, DL methods typically demonstrate superior accuracy and generalization potential, but their training demands pose practical deployment challenges. Conversely, ML and hybrid/ensemble approaches offer interpretable and resource-efficient alternatives, though their performance may vary considerably depending on the dataset characteristics and preprocessing procedures. The challenges identified, such as the difficulty of extracting reliable features from subtle or low contrast lesions, the risk of overfitting on small or imbalanced datasets, and the computational burden of training and deploying complex models, emphasize the need for ongoing research towards more generalized and interpretable models. Future research should focus on advancing transfer learning, multimodal integration, and optimization techniques that reduce computational load while enhancing robustness and interpretability. Strengthening clinical validation across diverse populations and imaging protocols will be essential to ensure diagnostic systems that are not only accurate, but also clinically practical and trustworthy. Ultimately, future efforts should prioritize building systems that are not only high-performing in controlled experiments, but are also reliable, explainable, and feasible for integration into real clinical workflows. This step will be key to transforming current research outcomes into truly usable and trustworthy diagnostic tools.

## Figures and Tables

**Table 1:** Machine learning-based classification.

No.	Ref.	Datasets	Segmentation Method	Feature Extraction and Selection	Classification Methods	Result	Limitation
1	[16]	400 private mammogram images	Not applicable	Morphological, texture, and density features	Extreme Learning Machine (ELM)	Benign and malignant categories: Accuracy: 96.2%, sensitivity: 95.8%, and specificity: 96.6%	Manual extraction of features (such as morphological features), which can still be prone to human error or variability based on expert experience.
2	[17]	Mammographic Image Analysis Society (MIAS)	k-means clustering algorithm	Gray Level Co-occurrence Matrix (GLCM) and Gray Level Run Length Matrix (GLRLM)	SVM, RF, ANN, k-NN, Naive Bayes (NB), DT	Normal vs. Abnormal, and benign vs. malignant: SVM, RF, and Neural Networks showed the best performance	Reliance on the mini-MIAS dataset, which is relatively small, potentially limiting the generalizability of the results to larger datasets or more diverse populations.
3	[18]	DDSM dataset	Spectral clustering	Feature extraction: GLCM, Feature selection: Genetic Algorithm (GA)	SVM: classify regions as mass or non-mass	Sensitivity: 89.5%, Specificity: 91.2%, Accuracy: 90%	Spectral clustering method may struggle with complex mass boundaries and overlapping tissues, leading to reduced accuracy in highly heterogeneous breast images.
4	[19]	MIAS, DDSM	CNN optimized by the Grasshopper Optimization Algorithm (GOA)	Geometric features, texture features, statistical features. GOA is used for feature selection	SVM	Sensitivity: 96%, Specificity: 93%, Accuracy: 92%	The learning time is relatively high due to the large number of iterations required for optimization
5	[20]	MIAS, INbreast	Not applicable	Feature extraction: ResNet18 extracts 512 features from each mammogram Feature selection: PSO, DFOA, CSOA	Weighted K-Nearest Neighbor (wKNN)	Benign and malignant classification: MIAS: accuracy: 84.35% using CSOA-wKNN INbreast: accuracy: 83.19% using CSOA-wKNN	The computational complexity of metaheuristic algorithms, particularly DFOA, which requires significant parameter tuning and exhibits a slower convergence rate compared to PSO and CSOA.
6	[21]	MIAS, DDSM, INbreast	Not applicable	Feature extraction: Fast discrete curvelet transform (FDCT-WRP) Feature selection: PCA and LDA	Extreme Learning Machine (ELM)	Benign vs. malignant classification: Accuracy achieved on MIAS: 100%. Accuracy on DDSM: 98.94%. Accuracy on INbreast: 98.76%.	ELM model’s complexity can be an issue, particularly regarding the computational cost involved in feature extraction and optimization
7	[22]	DDSM	Not applicable	Feature Extraction: Intensity-based Features, texture-based Features, shape-based Features Feature Selection: Biogeography-Based Optimization (BBO)	Adaptive Neuro-Fuzzy Inference System (ANFIS), ANN	Benign vs. malignant classification: Accuracy: 98.92% Sensitivity: 99.10% Specificity: 98.72%	The model was tested on a relatively small dataset, and the study did not explore other advanced classifiers or deeper neural networks
8	[23]	CBIS-DDSM	RoI based U-Net	Deep learning-based Scale Invariant Feature Transform (SIFT)	Fuzzy Decision Tree (FDT)	Normal, Benign, and malignant: Accuracy: 99.2%, Sensitivity: 95.36%, specificity: 97.41%	Relies on handcrafted feature (SIFT), which may limit performance, generalizability restricted since only tested on CBIS-DDSM.
9	[24]	MIAS, DDSM, BCDR	Not applicable	Feature extraction: Discrete Wavelet Packet Transform (DWPT). Feature selection: PCA and Weighted Chaotic Salp Swarm Algorithm (WC-SSA)	Kernel Extreme Learning Machine (KELM)	Normal vs. Abnormal Classification: MIAS dataset: Accuracy of 99.62%. DDSM dataset: Accuracy of 99.92%. Benign vs. Malignant Classification: MIAS dataset: Accuracy of 99.28%. DDSM dataset: Accuracy of 99.63%.	The combination of wavelet-based feature extraction and the complex WC-SSA optimization adds computational overhead
10	[25]	DDSM	Not applicable	Feature Extraction: Intensity-based Features, texture-based Features, and shape-based Features Feature Selection: Genetic Algorithm (GA)	AdaBoost, RF, and DT.	Benign and malignant classification: AdaBoost achieved: Accuracy: 96.15%. Random Forest achieved: Accuracy: 92.70%	The use of Genetic Algorithms, along with ensemble methods, increases the computational complexity of the model
11	[26]	MIAS, and Digital Mammography DREAM Challenge Dataset	Not applicable	Feature Extraction: Statistical features. Feature Selection: Best First Search	k-NN, Decision Trees (J48, Random Forest, Random Tree).	Normal vs. abnormal classification: Adaboosting J48, Decision Tree, and Random Forest: Accuracy: 100%. AUC: 1.000 (MIAS dataset).	Although data augmentation was applied, the number of abnormal samples was still limited compared to normal ones
12	[27]	MIAS Dataset	Thresholding-based segmentation	Feature Extraction: Gray Level Co-occurrence Matrix (GLCM) Feature Selection: Genetic Algorithm (GA)	Modified SVM	Accuracy: 96.34%. Sensitivity: 94.28% (ability to correctly detect malignant cases).	The study used a relatively small dataset (322 images), which could limit the generalizability of the results when applied to larger or more diverse datasets

**Table 2:** Deep learning-based classification approaches.

No.	Ref.	Datasets	Segmentation Method	Feature Extraction	Classification Methods	Result	Limitation
1	[28]	CBIS-DDSM, INbreast	Otsu Thresholding	CNN: DenseNet-169	Deep Location Soft-Embedding-Based Network-Regional Scoring (DLSEN-RS)	Benign, and malignant: INbreast dataset: accuracy: 91.5%. CBIS-DDSM dataset: accuracy: 89.4%.	A limitation of the DLSEN-RS model is the challenge of determining the optimal k value. If “k” is too small, important information might be missed, and if too large, redundant and irrelevant features may be selected, leading to reduced performance.
2	[29]	INbreast, CBIS-DDSM, BNS	Mask R-CNN	YOLOv5 and Mask R-CNN	Mask R-CNN	Benign, and malignant: False Positive Rate (FPR): 0.049% False Negative Rate (FNR): 0.029%	Computational complexity associated with training both YOLOv5 and Mask R-CNN
3	[30]	INbreast and HX dataset (private dataset)	Otsu segmentation	Pretrained DenseNet model	Deep Multiscale Multi-Instance Networks	Benign, and malignant: INbreast dataset: Accuracy: 93.2%. HX dataset: Accuracy: 0.872	The choice of the k value, which determines the number of patches to be selected for classification. A suboptimal value of k could lead to the inclusion of irrelevant regions, reducing performance.
4	[31]	INbreast, CBIS-DDSM	Otsu segmentation	DenseNet, ImageNet	DenseNet, ImageNet.	Benign, and malignant: INbreast dataset: Accuracy: 91.6% CBIS-DDSM dataset: Accuracy: 83.9%	Dividing the image into many smaller regions and calculating probabilities for each increases the time and computational resources needed for processing.
5	[32]	INbreast Dataset	Not applicable	YOLO-V4	ResNet, VGG, and InceptionNet.	Benign, and malignant: Inception-V3 accuracy: 98%	The need for significant computational resources for processing large mammograms and handling cropped slices, which may slow down real-time applications.
6	[33]	MIAS, CBIS-DDSM	Not applicable	CNN	Various CNN architectures (VGGNet, ResNet, and GoogLeNet).	AUC of 0.932 for mass and calcification and 0.84 for malignant and benign.	Computational complexity of fusing multi-view data.
7	[34]	DDSM, Hanoi Medical University (HMU) dataset	Not applicable	ResNet-34,	ResNet-34	Normal, benign, and malignant: macAUC of 0.766	The availability of annotated mammogram datasets, which restricts the fine-tuning process and may limit the generalization of the model.
8	[35]	Unknown mammogram dataset	Morphological clustering, dilation and erosion	Gray Level Run Length Matrix (GLRLM)	Kernel-Based Convolutional Neural Network (KBCNN)	Benign and malignant accuracy: 95%	The computational cost is associated with using KBCNN, which may hinder real-time processing.
9	[36]	INbreast Dataset	Not applicable	ECA-Net50 model	Efficient Channel Attention (ECA-Net50)	Benign and malignant categories: Accuracy: 92.9%. Precision: 88.3%.	The study relies on the INbreast dataset, which is relatively small compared to large-scale datasets.
10	[37]	INbreast Dataset	Mask R-CNN (Region-based Convolutional Neural Network)	Not applicable	Mask R-CNN framework	Benign and malignant: True Positive Rate (TPR) of 0.936 with a standard deviation of 0.063	The small size of the INbreast dataset, which may not provide enough diversity to fully train modern deep learning models like Mask R-CNN.
11	[38]	CBIS-DDSM, INbreast	Not applicable	Parallel Feature Extraction Stem (PFES), Dense Connection Blocks (DCB) and Inception Blocks (IB)	YOLO-based multiscale parallel CNN architecture.	INbreast Dataset: Accuracy: 98.72%	YOLO model can be biased toward smaller lesions due to its default loss function
12	[39]	510 digital mammogram images collected from Erbil hospital (Zheen)	Mask R-CNN	ResNet152V2	CNN: ResNet152V2, and Mask R-CNN	ResNet152V2: Accuracy: 100% for classifying breast density types and distinguishing normal or abnormal tissue.	The difficulty in detecting tumors in extremely dense breasts (types C and D), which may affect the system’s accuracy in these specific cases.
13	[40]	Radiological Society of North America (RSNA)	Not applicable	CNN-based architectures (ResNet18, ResNet34, ResNet152, EfficientNetB0, MaxViT)	Multiple pre-trained models (ResNet, EfficientNet, and MaxViT)	Normal and abnormal categories: ResNet18: Accuracy: 94% EfficientNetB0: Accuracy: 95% MaxViT: Accuracy: 89%	The scaling of images to 256 × 256 and 512 × 512 pixels, which might reduce classification performance
14	[41]	CBIS-DDSM Dataset	Not applicable	DenseNet	Enhanced Recurrent Neural Network (E-RNN)	Benign and malignant categories: Accuracy: 95% Matthews Correlation Coefficient (MCC): 91%	Difficulty in balancing data across institutions in federated learning setups and the challenge of optimizing communication between clients and the central server.
15	[42]	DDSM Dataset	Not applicable	Vision transformers (ViT)	Vision transformers	Benign and malignant categories: 1.00 ± 0 of accuracy	Vision transformers require high computational resources, particularly in large variants like ViT-large.
16	[43]	CBIS-DDSM, INbreast, and MIAS	The Probabilistic Anchor Assignment (PAA) algorithm, an anchor-free object detection approach	EfficientNet-B3 as the backbone network	Faster R-CNN and PAA	AUC for ROI classifier: 0.889.	The non-maximum suppression (NMS) and weighted box fusion (WBF) algorithms used in post-processing may still miss detections in dense breasts.
17	[44]	INbreast and private dataset	Square small patches (sliding window) are generated from the region	Not applicable	Faster R-CNN	Mean Average Precision (mAP): 0.94	Although the model achieves high accuracy, the use of small patches might still miss certain lesions if not appropriately handled during training.
18	[45]	INbreast Dataset	Not applicable	DenseNet and AlexNet	DenseNet and AlexNet	Benign and malignant categories: DenseNet: accuracy: 99.8%. AlexNet: accuracy: 98.8%	The study does not address potential biases in the dataset that could arise from its small size and class imbalance.
19	[46]	MIAS Dataset	Not applicable	Not applicable	Nasnet-Mobile: classifying mammographic images into benign or malignant categories. Modified ResNet50 (MOD-RES): fine-tuned for classifying breast masses	MOD-RES accuracy: 89.5%. Nasnet-Mobile: accuracy: 70%	The models are trained on a relatively small dataset, which might not generalize well to larger and more diverse populations
20	[47]	MIAS, DDSM, INbreast Dataset	Not applicable	CNN models: InceptionResNet-V2, Inception-V3, VGG-16, VGG-19, GoogLeNet, ResNet-18, ResNet-50, and ResNet-101	Transferable Texture Convolutional Neural Network (TTCNN)	Benign and malignant categories: DDSM: Accuracy: 99.08%. INbreast: Accuracy: 96.82%. MIAS: Accuracy: 96.57%	Though the proposed method shows significant improvement, there is still room for enhancing accuracy, especially in detecting smaller tumors at very early stages.
21	[48]	CBIS-DDSM	Not applicable	CNN: VGG16 network	BreastNet18 model, which is a fine-tuned version of VGG16.	Benign and malignant: Training accuracy: 96.72% Validation accuracy: 97.91% Test accuracy: 98.02%	The relatively small size of the dataset could lead to overfitting despite data augmentation
22	[49]	INbreast	Not applicable	CNN: DenseNet121 and MobileNet	CNN: DenseNet121 and MobileNet	DenseNet Accuracy: 96.34% MobileNet Accuracy: 95.12%	Although DenseNet provides excellent performance, it is computationally expensive. While MobileNet is more efficient, its performance is slightly lower.
23	[50]	CBIS-DDSM, INbreast, and private dataset collected from University Hospital “Paolo Giaccone,” Palermo, Italy	Yolo-based model	Yolo model	Yolo-based model	Benign and malignant: CBIS-DDSM dataset: mAP (mean Average Precision) of 0.498. INbreast dataset: mAP of 0.835	The proprietary dataset is heavily imbalanced, with 82.4% of the lesions being malignant, which may affect the model’s ability to generalize well

**Table 3:** Hybrid/ensemble-based classification approaches.

No.	Ref.	Datasets	Segmentation Method	Feature Extraction	Classification Methods	Result	Limitation
1	[52]	DDSM dataset	Not applicable	CNN: AlexNet	BreastNet + SVM	Benign, and malignant categories: Accuracy: 99.16% Sensitivity: 97.13% Specificity: 99.30%	The model’s performance may be impacted by the choice of optimizers, with varying results based on hyperparameter tuning.
2	[53]	CBIS-DDSM, INbreast dataset	Not applicable	Not applicable	Attention mechanisms + CNN architectures (ResNet50, DenseNet169, RegNetX064)	CBIS-DDSM dataset: DenseNet169 + Attention Module: AU-ROC of 0.79. INbreast dataset: DenseNet169 + Squeeze and Excitation (SE): AU-ROC of 0.88	Attention improved performance inconsistently across different datasets and abnormality types. Additionally, complex models with higher pooling tended to overfit on smaller datasets, and certain attention mechanisms such as convolutional bottleneck attention module (CBAM) were harder to optimize.
3	[54]	MIAS and DDSM dataset	Thresholding and region growing method	GLCM, shape and margin features.	An ensemble classifier model including KNN, bagging, and EigenClass algorithms	Normal, benign, or malignant categories: DDSM’s accuracy: 93.26%. MIAS’s accuracy: 91%.	Computational complexity due to the ensemble classification and feature weighting algorithms.
4	[55]	MIAS Dataset	A Gabor filter	CNN model	CNN + Extreme Learning Machine (ELM)	Benign and malignant categories: The hybrid CNN-ELM model Accuracy: 86% on the MIAS dataset.	The model was tested on a relatively small dataset (MIAS dataset with 322 images), which may limit its generalizability to larger datasets.
5	[56]	MIAS, INbreast, BCDR Dataset	Not applicable	Probabilistic Principal Component Analysis (PPCA)	Naïve Bayes + Firefly Binary Grey Optimization (FBGO), Transfer Convolutional Neural Network (TCNN) + Moth Flame Lion Optimization (MMFLO)	Benign and malignant categories: Naïve Bayes + FBGO (MIAS dataset): Accuracy: 96.3% TCNN + MMFLO (MIAS dataset): Accuracy: 98%	The high computational complexity associated with the ensemble model, especially when combining both Naïve Bayes and TCNN models.
6	[57]	MIAS, INbreast, CBIS-DDSM Dataset	Not applicable	CNN architecture: VGG16, VGG19, ResNet50, and DenseNet121	CNN hybrid approach: VGG16, VGG19, ResNet50, DenseNet121.	Normal, Benign, and malignant categories: MIAS’s accuracy: 98.70% Inbreast’s accuracy: 98.83%.	Computational complexity due to the combination of multiple CNN models. Additionally, there were slight challenges in discriminating against malignant cases compared to normal and benign cases.
7	[58]	CBIS-DDSM Dataset	Not applicable	Pre-trained models (ResNet-50, EfficientNet-B5, and Xception)	ResNet-50 + Xception + EfficientNet-B5.	Mass/calcification classification: accuracy: 91.36%. Benign/malignant classification: accuracy: 76.79%.	Computational complexity due to the combination of multiple CNN models.
8	[59]	VinDr-Mammo, DDSM, CMMD, CDD-CESM, BMCD, RSNA	YOLOX	The features for classification are extracted using two CNN architectures: EfficientNet and ConvNeXt	EfficientNet-B7 + ConvNeXt-101	VinDr-Mammo’s accuracy: 90% CMMD’s accuracy: 92% BMCD’s accuracy: 92%	The primary limitation of the study lies in the reliance on gradCAM for visualizing the important regions of the ROIs, which may sometimes produce noisy or incomplete heat maps.
9	[60]	Breast Cancer Digital Repository (BCDR) dataset.	Not applicable	Pre-trained classical neural networks (ResNet18)	Combination of pre-trained classical models (ResNet18) + quantum neural networks	Normal and abnormal categories: 84% of accuracy.	The study notes that quantum devices are still in the early stages of development (NISQ era), and the results from real quantum devices showed slightly lower accuracy (81%) compared to the quantum simulator (84%).
10	[61]	MIAS Dataset	Not applicable	GLCM and statistical features	A hybrid model combining SVM for the first stage of classification (normal vs. abnormal) and ANN for the second stage (benign vs. malignant)	The hybrid SVM + ANN model: 99.4% of accuracy for normal vs. abnormal classification.	The use of only 160 mammograms for training and testing limits the generalizability of the model. The small dataset size could introduce overfitting or bias in the model’s performance.
11	[62]	MIAS Dataset	Not applicable	Convolutional Neural Network (CNN) + Graph Convolutional Network (GCN)	CNN + Graph Convolutional Network (GCN)	Sensitivity: 96.20 ± 2.90% Specificity: 96.00 ± 2.31% Accuracy: 96.10 ± 1.60%	The dataset was imbalanced, with fewer abnormal images (113) compared to normal images (209), which may affect the model’s generalizability
12	[63]	IRMA Dataset	Not applicable	Statistical Features, Local Binary Pattern (LBP) Features, Taxonomic Features, and Dynamic Features	SVM + Emotional Learning inspired Ensemble Classifier (ELiEC)	Accuracy: 80.54%.	While hybrid features improved accuracy, the margin of improvement was around 2–3%, which, while notable, may still leave room for optimization.
13	[64]	BCDR, MINI-MIAS, DDSM, INbreast Dataset	Not applicable	Deep convolutional neural networks (CNN): VGG-11, ResNet-164, DenseNet121, and Inception V4	Combination of fuzzy ensemble modeling+deep CNNs (VGG-11, ResNet-164, DenseNet121, and Inception V4)	Normal, benign, and malignant categories: accuracy of 99.32%.	The use of multiple CNNs in an ensemble increases computational complexity.
14	[65]	MIAS, DDSM Dataset	Not applicable	Feature Extraction: GLCM Feature Selection: semi-supervised K-means clustering algorithm	AdaBoost combined with multiple base classifiers: Decision Tree (DT), k-Nearest Neighbors (KNN), Support Vector Machine (SVM), and hybrid SVM-KNN	Benign and malignant classification: DDSM Dataset: AdaBoost with Hybrid SVM-KNN: 90.625% of accuracy.	The study relies on manual ROI segmentation, which may limit its applicability in fully automated systems.
15	[66]	MIAS, INBreast, CBIS-DDSM	IEUNet++	Not applicable	Hybrid deep learning IEUNet++	Normal, benign, malignant: INBreast: Accuracy: 99.87%, sensitivity: 99.77%, specificity: 0.998	Computationally expensive due to ensemble of InceptionResNetV2 + EfficientNetB7.

**Table 4:** Performance ranges of ML, DL, and hybrid methods in breast cancer classification.

Method Approach	Accuracy Range	Sensitivity Range	Precision Range	Specificity Range
Machine Learning	82.42–100% (normal and abnormal class)	86.67–99.1%	82.42–83.87%	83.33–98.72%
Deep Learning	70–100% (normal and abnormal class)	X	88.3–99.16%	X
Hybrid	74.96–99.87% (normal, benign, malignant)	96.2–99.77%	X	96–99.8%

**Table 5:** Representative high-performing models by classification approach.

Approaches	Datasets	Pre-Processing	Feature Extraction	Feature Selection	Optimization	Classification	Accuracy
Machine learning [21]	MIAS, DDSM, INbreast	X	Fast discrete curvelet transform (FDCT-WRP)	Principal Component Analysis (PCA) and Linear Discriminant Analysis (LDA)	Modified Particle Swarm Optimization	Extreme Learning Machine (ELM)	100% (benign and malignant class)
Deep learning [42]	DDSM	Data balancing (augmentation), color jitter, gamma correction, salt and pepper noise	Vision transformers and pretrained weights from ImageNet	X	X	Vision transformer	100% (benign and malignant class)
Hybrid/Ensemble [66]	MIAS, CBIS-DDSM, INbreast Dataset	Segmentation: IEUNet++	X	X	X	InceptionResNet + EfficientNetB7 + UNet (IEUNet++)	99.87% (normal, benign, and malignant)

**Table 6:** Mammogram Datasets.

Datasets	Total Images	Lesion Type	Image Category	Usage in This Review Article	Remarks
MIAS/MINI-MIAS	322	All kind	Normal, Benign, and Malignant	23 studies	Classic, widely used despite small size
DDSM	10,480	Mass, calcification	Normal, Benign, and Malignant	23 studies	One of the earliest large datasets, image quality is relatively low.
CBIS-DDSM	3012	Mass, calcification	Benign, Malignant	14 studies	Curated, more consistent version of DDSM
INbreast	410	All kind	Normal, Benign, And Malignant	21 studies	High-quality, pixel-level annotation
BCDR	7315	All kind	Normal, Cancer	4 studies	Includes RoI annotation

## Data Availability

No new data were created or analyzed in this study. Data sharing is not applicable to this article.

## Abbreviations

The following abbreviations are used in this manuscript:

CAD	Computer Aided-Detection
ML	Machine Learning
DL	Deep Learning
SVM	Support Vector Machine
CNN	Convolution Neural Network
PRISMA	Preferred Reporting Items for Systematic Reviews and Meta-Analyses
RoI	Region of Interest
GLCM	Gray Level Co-occurrence Matrix
YOLO	You Only Look Once
IoU	Intersection over Unit
AUC	Area Under Cover
ROC	Receiver Operating Characteristic
ELM	Extreme Learning Machine
KNN	k-Nearest Neighbors
MFO	Moth Flame Optimization
PSO	Particle Swarm Optimization
ECA	Efficient Channel Attention
MLO	Mediolateral-Oblique
CC	Cranio-Caudal
